# Assessing the Impact: The Damage Index for Antiphospholipid Syndrome in the Context of Other Autoimmune Diseases and Cardiovascular Risk Factors

**DOI:** 10.7759/cureus.79849

**Published:** 2025-02-28

**Authors:** João Faia, Eulália Antunes, Ana Luisa Marques, Pedro Bem Haja, Graziela Carvalheiras

**Affiliations:** 1 Internal Medicine, Unidade Local de Saúde da Região de Aveiro, Aveiro, PRT; 2 Internal Medicine, Hospital de Braga, Braga, PRT; 3 Internal Medicine, Centro Hospitalar de Entre Douro e Vouga, Santa Maria da Feira, PRT; 4 Department of Education and Psychology, Center for Health Technology and Services Research at the Associated Laboratory RISE (CINTESIS@RISE) University of Aveiro, Aveiro, PRT; 5 Clinical Immunology Unit, Unidade Local de Saúde de Santo António, Porto, PRT

**Keywords:** antiphospholipid syndrome, cardiovascular disease, cardiovascular risk factor, damage index, damage index for antiphospholipid syndrome (diaps)

## Abstract

Background

Antiphospholipid syndrome (APS) is a chronic autoimmune disorder characterized by thrombotic events and organ damage, often leading to significant morbidity and mortality. The Damage Index for Antiphospholipid Syndrome (DIAPS) was developed to quantify irreversible damage in these patients, providing a tool for better disease management.

Objectives

This study investigates the long-term accumulation of organ damage in patients with thrombotic APS. Specifically, it examines how damage severity differs between primary APS (PAPS) and secondary APS (SAPS) and how traditional cardiovascular risk factors contribute to disease progression. Understanding these interactions may help refine patient management strategies.

Methods

A retrospective analysis of 141 patients diagnosed with thrombotic APS was conducted using medical records. The DIAPS score was calculated for each patient, and its association with autoimmune comorbidities and cardiovascular risk factors was analyzed through statistical modeling.

Results

Among the 141 APS patients (86% female, mean age 52 years), systemic lupus erythematosus was the most frequent associated autoimmune disease (92%). Arterial hypertension was present in 39% of cases, dyslipidemia in 28%, and type 2 diabetes in 10%. Patients with SAPS had significantly higher DIAPS scores than those with PAPS (p=0.044). Hypertension and diabetes were linked to increased organ damage, while dyslipidemia influenced the relationship between APS-related autoimmunity and cumulative damage.

Conclusions

Patients with secondary APS experience more severe long-term damage compared to those with primary APS. Additionally, cardiovascular risk factors, particularly hypertension and diabetes, worsen disease progression. These findings underscore the need for a multidisciplinary approach that integrates autoimmune disease management with cardiovascular risk control to prevent irreversible complications in APS patients.

## Introduction

Background

Antiphospholipid syndrome (APS) is a complex autoimmune disorder characterized by the persistent presence of antiphospholipid antibodies (aPL) and a wide spectrum of clinical manifestations, such as venous and arterial thrombosis, recurrent miscarriages, and organ damage [1,2]. In severe cases, APS may progress to catastrophic APS (CAPS), leading to multiorgan failure and increased mortality, thereby significantly compromising patients' quality of life [3,4].

Given the chronic and progressive nature of APS, quantifying cumulative organ damage is essential for effective long‐term management. In response to this need, the Damage Index for Antiphospholipid Syndrome (DIAPS) was developed as a comprehensive tool to assess irreversible damage resulting from both thrombotic and non‐thrombotic manifestations of APS [5,6]. Derived from the Systemic Lupus International Collaborating Clinics/American College of Rheumatology Damage Index (SLICC/ACRDI) used in systemic lupus erythematosus (SLE), DIAPS incorporates additional criteria tailored specifically to the unique clinical profile of APS [7]. This index evaluates damage across multiple organ systems (including cardiovascular, renal, pulmonary, neurological, and others) and has shown potential as a robust marker of disease progression over extended time periods. Initially validated in Latin American cohorts, subsequent studies have confirmed its utility in more diverse populations [5-6,8-9].

Objectives

Although DIAPS is available, the literature offers limited direct comparisons between primary APS (PAPS) and secondary APS (SAPS) associated with other autoimmune diseases, and the influence of traditional cardiovascular risk factors (CVRF), such as arterial hypertension, dyslipidemia, and type 2 diabetes mellitus on long‐term damage remains unclear. Previous studies have examined DIAPS in various contexts; however, a gap exists in understanding how these factors interact to affect irreversible organ damage. Therefore, our study addresses the following research question: Do APS patients with secondary autoimmune conditions exhibit significantly higher DIAPS scores compared to those with primary APS, and is this difference further exacerbated by the presence of cardiovascular risk factors? In other words, we hypothesize that SAPS patients will demonstrate greater cumulative organ damage than PAPS patients, particularly when concomitant CVRF are present. By testing this hypothesis, our study aims to provide novel insights that can enhance risk stratification, inform treatment decisions, and improve long‐term surveillance in APS management.

## Materials and methods

Study design and population

This retrospective, descriptive, single-center study was conducted in a specialized tertiary care hospital with a dedicated autoimmune disease (AID) clinic. Electronic medical records of patients with confirmed or suspected antiphospholipid syndrome were analyzed according to the Revised Sapporo Classification Criteria (Sydney, 2006) [1]. The study period encompassed all available records from as early as 1987 until December 2023. Patients with thrombotic APS (APS-T) who were alive at the time of analysis were included. Exclusion criteria were as follows: patients not meeting the Revised Sapporo Classification Criteria, patients with isolated obstetric APS manifestations, patients with incomplete clinical data, deceased patients (to prevent survival bias).

Data collection

Data were extracted from electronic medical records by three independent investigators following a standardized protocol. Predefined criteria were used to ensure consistency, and any discrepancies were resolved by consensus. The variables collected included demographic data, associated autoimmune diseases, cardiovascular risk factors, and organ damage assessed using the Damage Index for Antiphospholipid Syndrome. DIAPS was calculated according to the recommendations of Amigo et al. [5] and comprises 37 items across various organ systems (e.g., cardiovascular, renal, pulmonary, neurological), with a total score ranging from 0 (no damage) to 37 points.

Statistical analysis

Statistical analyses were performed using R software (version 4.3.3; R Foundation for Statistical Computing, Vienna, Austria). The normality of continuous variables was assessed using the Shapiro-Wilk test. Welch's t-test was used to compare mean DIAPS scores between patients with PAPS and SAPS. Effect sizes were expressed using Hedge's g with a 95% confidence interval. Additionally, linear regression models were employed to assess the association between DIAPS scores and the presence of CVRF. Simple effects analyses were conducted to evaluate the moderating effect of CVRF on the relationship between DIAPS and associated autoimmune diseases. A significance level of p<0.05 was used for all tests.

Ethical considerations

This study adhered to the ethical principles outlined in the Declaration of Helsinki. Due to the retrospective nature of the analysis, informed consent was waived, and all data were anonymized to ensure participant privacy.

## Results

**Figure 1:**
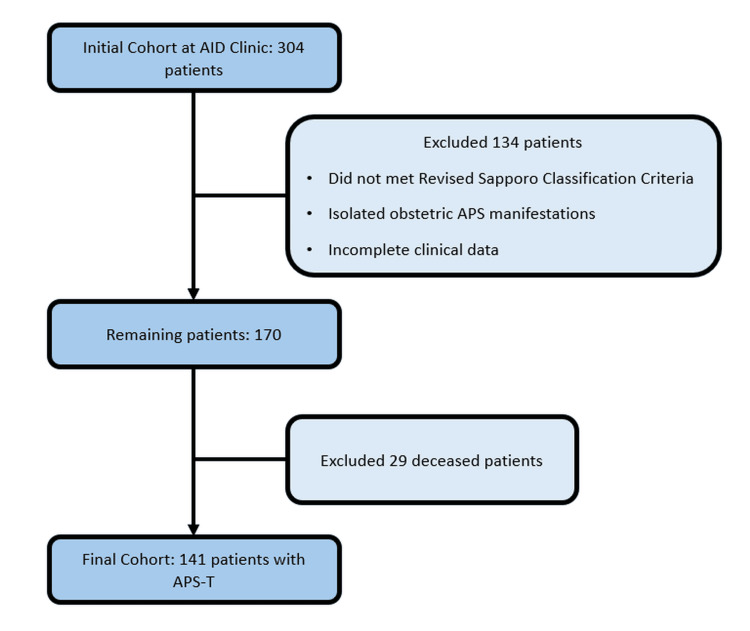
Flow diagram of patient selection AID - autoimmune disease; APS-T - thrombotic antiphospholipid syndrome

Out of the initial cohort of 304 patients followed at the AID clinic, 134 were excluded because they did not meet the Sapporo classification criteria for APS, did not present APS-T, or had incomplete clinical data. Among the remaining 170 patients diagnosed with APS-T, an additional 29 were excluded due to mortality (Figure 1).

Demographic characteristics and associated autoimmune conditions

In this cohort of 141 APS patients, 121 (86%) were female, with an average age of 52 years. Among these patients, 75 (53%) had an AID, with systemic lupus erythematosus (SLE) being the most common, affecting 69 out of the 75 patients (92%). Table 1 summarizes the other AIDs associated with APS in the study population.

**Table 1:** Distribution of autoimmune diseases associated with APS in the study population APS - antiphospholipid syndrome

Autoimmune disease (AID)	n	%
Systemic lupus erythematosus	69	92
Sjögren's syndrome	2	2.7
Rheumatoid arthritis	2	2.7
Systemic sclerosis	1	1.3
Autoimmune hepatitis	1	1.3

**Figure 2:**
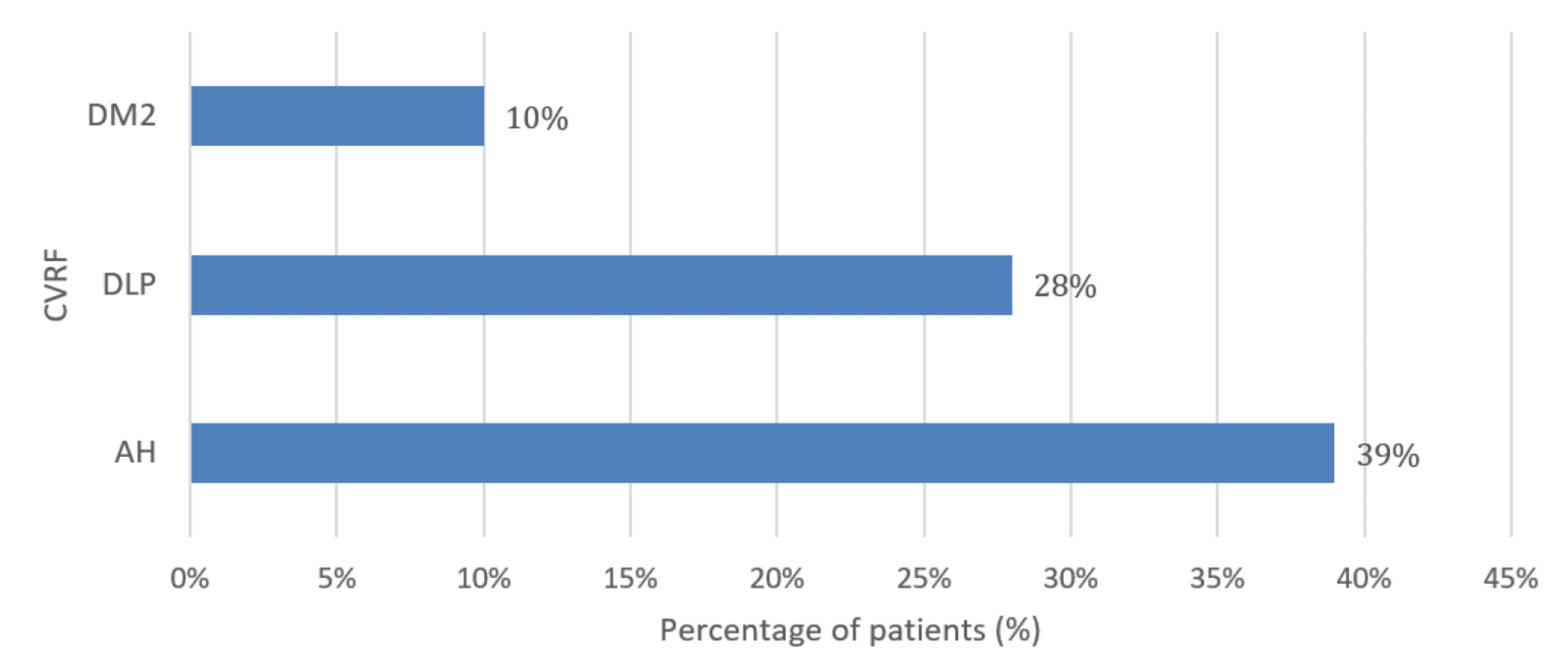
Distribution of CVRF in APS patients APS - antiphospholipid syndrome; AH - arterial hypertension; CVRF - cardiovascular risk factors; DLP - dyslipidemia; DM2 - type 2 diabetes mellitus

Regarding CVRF, the most prevalent were arterial hypertension (AH), found in 55 patients (39%), dyslipidemia (DLP) in 39 patients (28%), and type 2 diabetes mellitus (DM2) in 14 patients (10%). These three CVRF were the primary ones evaluated in the present study. The distribution of these CVRF is visually represented in Figure 2.

Overall assessment of DIAPS

**Figure 3:**
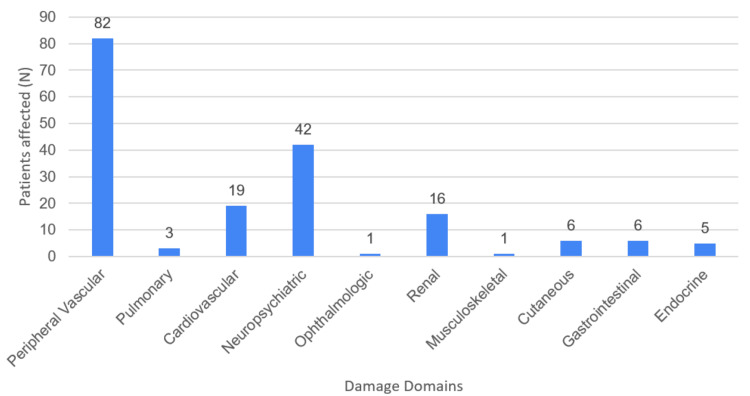
Organ-specific damage distribution

The DIAPS assessment revealed a heterogeneous distribution of damage across various organ systems. Peripheral vascular damage was the most commonly affected domain, observed in 82 patients. Neuropsychiatric damage was the second most frequent, affecting 42 patients, followed by cardiovascular and renal damage, affecting 19 and 16 patients, respectively. Other forms of damage, including pulmonary, musculoskeletal, endocrine and ophthalmologic, were less prevalent. A summary of the distribution across organ systems is presented in Figure 3. 

**Figure 4:**
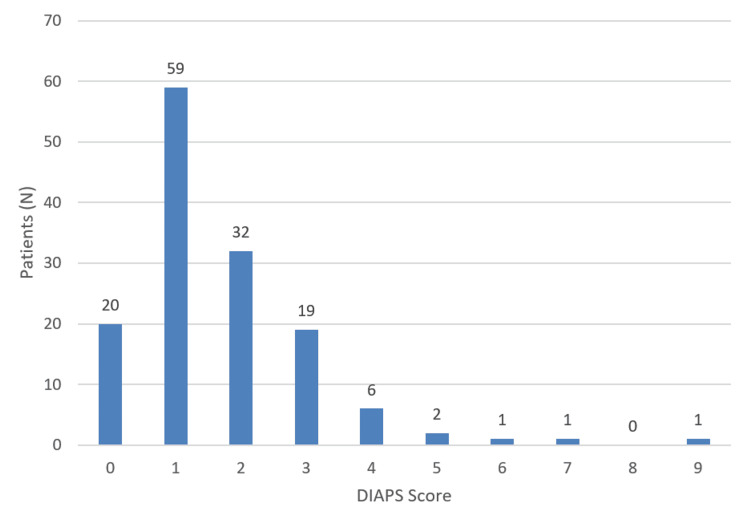
DIAPS score distribution DIAPS - Damage Index for Antiphospholipid Syndrome

The overall damage quantification in the cohort, based on DIAPS, yielded a mean score of 1.71 (range: 0-9). The lowest recorded score was 0, observed in 20 patients, indicating no irreversible damage in these cases. The highest recorded score was 9, with only one patient reaching this level of cumulative damage. The distribution of DIAPS scores is presented in Figure 4. The most common damage score was 1, observed in 59 patients, followed by a score of 2 in 32 patients. Higher scores were less frequent, with one patient each scoring 6, 7, and 9.

Correlation between DIAPS in PAPS, SAPS, and CVRF

**Figure 5:**
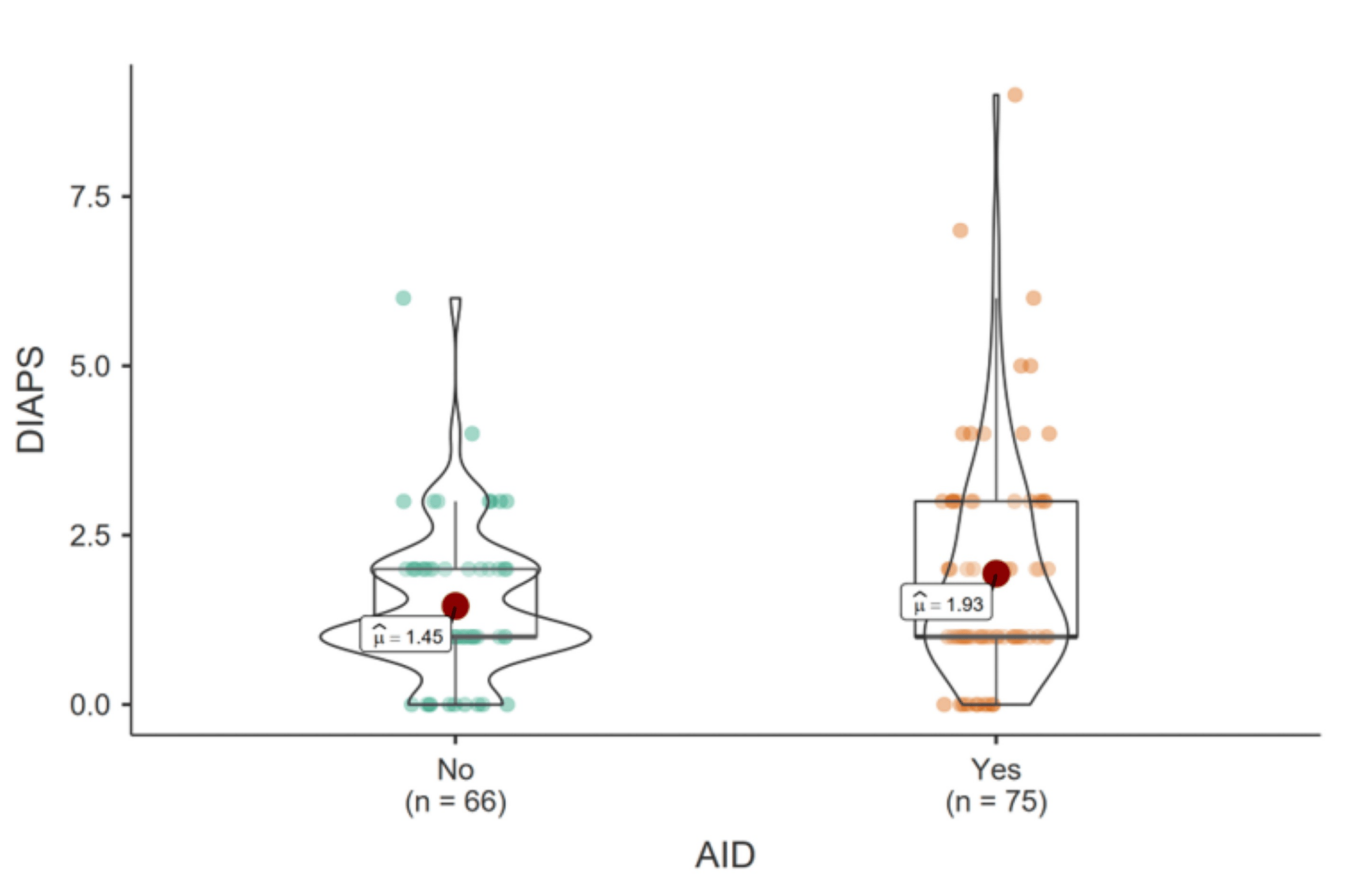
Distribution of DIAPS in the presence and absence of AID AID - autoimmune disease; DIAPS - Damage Index for Antiphospholipid Syndrome

Welch's t-test was performed to assess the relationship between DIAPS in the presence of AID associated with APS-T. The test revealed a significantly higher mean DIAPS in patients with SAPS compared to those with PAPS (Welch t(127.99)=-2.03, p=0.044, Hedge's g=-0.34, 95% CI: -0.67,-0.01; see Figure 5). The renal domain showed the greatest difference between groups, with higher renal damage observed in the SAPS group.

Subsequently, linear models were employed to assess whether the presence of CVRF also predicted higher DIAPS, whether the effect of AID remained significant, and whether CVRF moderated the relationship between AID and DIAPS.

**Figure 6:**
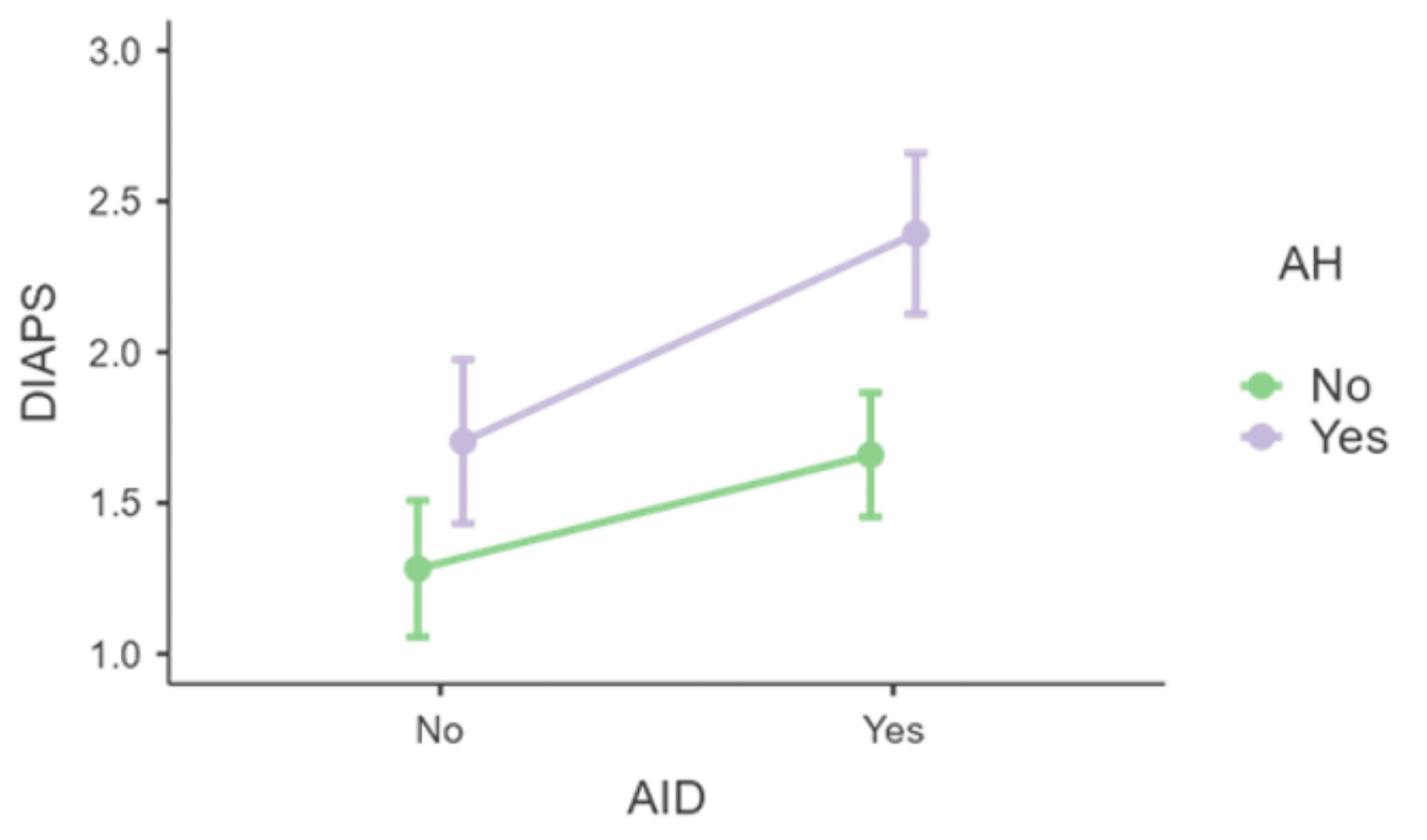
Distribution of DIAPS in the presence or absence of AID and AH AID - autoimmune disease; AH - arterial hypertension; DIAPS - Damage Index for Antiphospholipid Syndrome

The presence of AH was associated with an increase in DIAPS (F(1,137)=5.599, p=0.019, η²p=0.039). Although the effect of AID remained significant, further analysis of the simple effects of AID with AH as a moderator revealed that the significant difference in DIAPS between the presence and absence of AID was only observed when AH was present (F(1,137)=3.279, p=0.052; see Figure 6).

**Figure 7:**
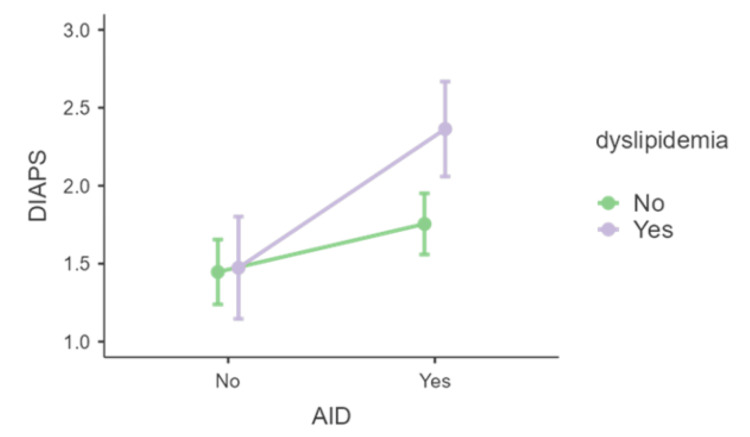
Distribution of DIAPS in the presence or absence of AID and dyslipidemia AID - autoimmune disease; DIAPS - Damage Index for Antiphospholipid Syndrome

Regarding DLP, its presence did not significantly alter DIAPS values (p>0.233). However, an analysis of the simple effects of AID with DLP as a moderator indicated that the significant difference in DIAPS between the presence and absence of AID was only observed when DLP was present (F(1,137)=3.98, p=0.048), and not when it was absent (F(1,137)=1.159, p=0.284, see Figure 7).

**Figure 8:**
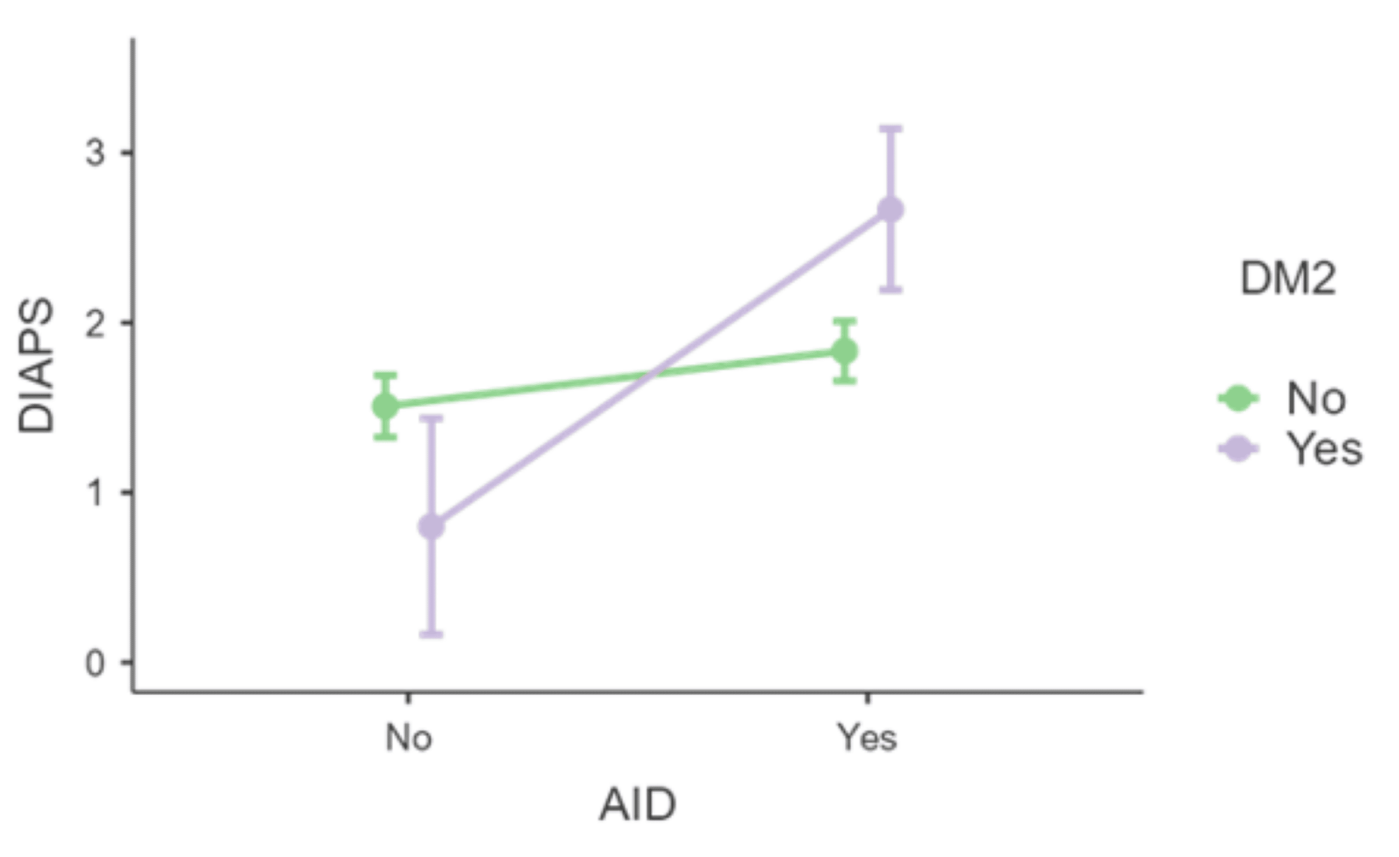
Distribution of DIAPS in the presence or absence of AID and DM2 AID - autoimmune disease; DIAPS - Damage Index for Antiphospholipid Syndrome; DM2 - type 2 diabetes mellitus

Lastly, for the presence of DM2, the analysis of simple effects revealed that the pattern observed with other risk factors not only persisted but was amplified, with a significant difference in DIAPS between the presence and absence of AID only occurring in the presence of DM2 (F(1,137)=3.98, p=0.048, see Figure 8).

## Discussion

The present study aimed to investigate the relationship between the DIAPS and various clinical factors, with particular emphasis on cardiovascular risk factors and associated autoimmune diseases in patients with thrombotic APS. Our findings indicate that patients with secondary APS exhibit significantly higher DIAPS scores than those with primary APS, a result that is in line with previous studies [8,10-12]. This suggests that the cumulative burden of additional autoimmune conditions, notably systemic lupus erythematosus, exacerbates irreversible organ damage in APS.

One of the key findings was that arterial hypertension (AH) emerged as a strong predictor of increased DIAPS scores. Our analysis demonstrated that the significant difference in damage between patients with and without AID was apparent only in the presence of AH. This observation supports the notion that hypertension may interact with APS pathophysiology to accelerate vascular injury. Similar observations have been reported in earlier studies [13,14], and our results reinforce the potential benefit of stricter blood pressure control in APS patients.

Additionally, while dyslipidemia did not independently alter DIAPS values, its presence moderated the relationship between AID and organ damage. Similarly, type 2 diabetes mellitus showed a pronounced moderating effect, with significant differences in DIAPS observed predominantly in diabetic patients with coexisting AID. These findings suggest possible mechanisms whereby dyslipidemia may enhance oxidative stress and endothelial dysfunction, and diabetes may contribute to vascular injury through chronic hyperglycemia, both of which are known factors in the progression of organ damage [15,16].

Clinically, these results underscore the importance of comprehensive CVRF management in APS patients, especially in those with secondary APS. Current guidelines for APS primarily focus on immunomodulatory and anticoagulant therapies; however, our findings advocate for an integrated approach that includes aggressive management of hypertension, dyslipidemia, and diabetes. This is particularly critical in SAPS patients, who are often managed primarily for their autoimmune disease while CVRF control might be underestimated. Notably, since the renal domain was one of the most affected in SAPS patients, future studies should also evaluate the role of chronic kidney disease (CKD) in exacerbating APS-related damage.

When comparing our findings with previous research, it is evident that our study extends the literature by including both PAPS and SAPS populations and by utilizing DIAPS, a tool that validated across diverse cohorts, to capture cumulative damage. Earlier studies focusing solely on PAPS have provided valuable insights into the impact of CVRF on APS [17]; however, our inclusion of SAPS patients offers novel perspectives on how underlying autoimmune conditions compound the risk.

Despite the strengths of our study, several limitations must be acknowledged. The single-center, retrospective design may limit the generalizability of our results due to potential selection bias and variability in clinical management across different institutions. Additionally, our reliance on electronic medical records raises the possibility of incomplete or missing data, which could affect the accuracy of DIAPS calculations. The exclusion of deceased patients to avoid survival bias may also lead to an underrepresentation of more severe cases, and the lack of longitudinal follow-up data restricts our understanding of how damage accrues over time.

In summary, our study provides compelling evidence that SAPS patients experience greater cumulative organ damage compared to PAPS patients and that CVRF - particularly, hypertension, dyslipidemia, and diabetes - play a significant role in moderating this damage. These findings highlight the need for a multidisciplinary approach that integrates rigorous CVRF management into the standard care of APS patients. Future prospective studies are warranted to confirm these associations and to determine whether targeted interventions for CVRF can improve long-term clinical outcomes in this population.

## Conclusions

The results of this study highlight the significant impact of autoimmune comorbidities and cardiovascular risk factors on the Damage Index for Antiphospholipid Syndrome in patients with thrombotic APS. Patients with secondary APS exhibited higher DIAPS scores compared to those with primary APS, suggesting a more severe disease course when additional autoimmune conditions are present. Moreover, traditional CVRF, such as arterial hypertension, dyslipidemia, and type 2 diabetes, were associated with increased long-term damage, underscoring the importance of managing these risk factors alongside APS treatment.

These findings reinforce the need for a multidisciplinary approach to APS management that addresses both autoimmune and cardiovascular aspects of the disease. In particular, our data suggest that more stringent control of CVRF may improve patient outcomes, which could prompt revisions to current management guidelines. Future research should prioritize multicenter and longitudinal studies to validate these findings across diverse populations and to elucidate the underlying biological mechanisms linking autoimmune diseases, CVRF, and cumulative organ damage. In the interim, clinicians are encouraged to integrate aggressive CVRF management into routine APS care, potentially leading to reduced irreversible damage and improved long-term outcomes.
